# Radiomics-based MRI model for differentiating ovarian cystadenoma and cystadenocarcinoma

**DOI:** 10.3389/fonc.2026.1839964

**Published:** 2026-08-27

**Authors:** Mohamed Muntasir Ramjaun, Palpasa Shrestha, Beebee Kaneeze Hasanayn Bhoodoo, Lisong Dai, Bulat Abdrakhimov, Zhen Huang, Xuechun Wang, Prajal Shrestha, Jun Chen

**Affiliations:** 1 Department of Radiology, Renmin Hospital of Wuhan University, Wuhan, Hubei, China; 2 Department of Pathology, Zhongnan Hospital of Wuhan University, Wuhan, Hubei, China; 3 Department of Cardiovascular Surgery, Renmin Hospital of Wuhan University, Wuhan, Hubei, China; 4 Department of Research and Development, United Imaging Intelligence, Shanghai, China; 5 School of Built Environment, Engineering and Computing, Leeds Beckett University, Leeds, United Kingdom

**Keywords:** machine learning, magnetic resonance imaging, ovarian cystadenocarcinoma, ovarian cystadenoma, radiomics

## Abstract

**Background:**

Accurate differentiation between benign and malignant ovarian tumors is crucial to ensure timely intervention for high-risk patients and minimizing overtreatment in others. Thus, the objective was to develop a radiomics-based machine learning model to differentiate between non-cancerous and cancerous lesions in ovaries.

**Methods:**

This retrospective study included 271 patients with ovarian cystadenocarcinoma and 266 patients with ovarian cystadenoma who underwent T2-weighted magnetic resonance imaging (MRI). Lesions were manually segmented, and 2286 radiomics features were extracted. Following dimensionality reduction, features were used to train machine learning models with different combinations of data scaling (min-max, Z-score, and quantile transformations) and classifiers (logistic regression and partial least squares discriminant analysis). In addition, a model combining radiomics features and cancer biomarker data (CA-125 and HE-4) was developed. Models were compared using the DeLong’s test and McNemar’s tests.

**Results:**

A total of 37 features were used for model development. The quantile logistic regression model exhibited the highest performance, achieving an AUC of 0.92, a sensitivity of 0.87, and a specificity of 0.83, which was significantly higher compared to all models except for Z-score logistic regression model. The inclusion of serum biomarkers (CA-125 and HE-4) significantly improved the performance of the model.

**Conclusion:**

T2-weighted MRI radiomics-based models accurately differentiated between benign and malignant epithelial ovarian tumors.

## 1 Introduction

Globally, over 300,000 women are diagnosed with ovarian cancer annually. Ovarian cancer continues to carry the highest mortality rate among all gynecologic malignancies (1, 2). Clinically, most patients present at an advanced stage, with only about 20-25% diagnosed at Stage I (3). This distribution largely explains the persistently poor five-year survival (4). Although historically labeled a “silent killer” (5), evidence shows that many women with early-stage disease report symptoms such as pelvic pain, bloating, and abdominal distension prior to diagnosis (6). The clinical challenge, therefore, lies not only in recognizing symptoms but in accurately evaluating adnexal masses once they are detected. Most adnexal lesions are benign, whereas ovarian malignancies remain highly lethal (7–10). Consequently, accurate differentiation between benign and malignant tumors is crucial to ensure timely intervention for high-risk patients and minimize overtreatment in others.

Pelvic ultrasound is the primary diagnostic tool for investigating an adnexal mass capable of differentiating between benign and malignant masses with high sensitivity and specificity (11, 12). Recent studies reported that the sensitivity and specificity of ultrasound and ultrasound-based models may be used as an alternative to other diagnostic modalities such as magnetic resonance imaging (MRI) in differential diagnosis and even ovarian cancer staging (11, 13, 14). Unfortunately, as ultrasound demands high experience and skillful hands, interobserver variability is of concern (15–17). Identification of suspicious adnexal masses by ultrasound that requires problem-solving is often followed by MRI examination for lesion characterization. The sensitivity of MRI in detecting malignant lesions has consistently been high, and its specificity has been improving over the years (18–20). Recently released Ovarian-Adnexal Reporting and Data System for MRI (O-RADS MRI) was developed to further improve risk stratification of benign and malignant tumors (20). Even so, interpretation remains partly qualitative and observer-dependent. Intermediate-risk categories can be challenging, and reader experience still matters. In addition to failure to interpret MRI scans, even misunderstanding definitions within O-RADS MRI can occur, which leads to false-positive results (21, 22).

Radiomics is an advanced image analysis approach that extracts large numbers of quantitative features such as intensity distribution, texture, and geometry that are difficult to assess by the human eye. These features are used to enhance predictive modeling and clinical decision support (23). It must be noted that predictive radiomics models are not used to replace physicians but rather complement and aid them in clinical decision-making. Although many recent studies in ovarian cancer reported encouraging diagnostic and prognostic performance of radiomics, they had methodological variability and limited reproducibility (24, 25). There is also an issue with over-fitting, which can occur in models trained on datasets with a very large number of features relative to sample size (26). Furthermore, multi-sequence protocols, heavy pre-processing, and unclear modeling steps may artificially inflate performance, making models invalid for application in the clinical setting (26, 27). Hence, there is growing interest in “direct” models that limit inputs and keep the workflow familiar while not significantly affecting the performance. The present study covers the development and assessment of such models to differentiate benign and malignant cystic epithelial ovarian lesions using T2-weighted MRI (T2WI) scans. In addition, a sub-study was performed to evaluate whether the inclusion of cancer biomarkers (CA-125 and HE-4) had a significant impact on the models’ performance.

## 2 Materials and methods

### 2.1 Study design and data collection

This study is based on a retrospective analysis of female patients at the Renmin Hospital of Wuhan University (Wuhan, China) between March 2015 and August 2023. The study was approved by the Ethics Committee of the Renmin Hospital of Wuhan University. Inclusion criteria were as follows: histopathological diagnosis of cystadenoma or cystadenocarcinoma (serous and mucinous subtypes) and T2WI (3.0T MRI) scans with available DICOM images. Patients with non-epithelial ovarian masses (e.g. endometriomas, dermoids), extra-ovarian pelvic masses (e.g. para-ovarian cyst, peritoneal inclusion cyst, hydrosalpinx) were excluded. Age and cancer biomarker levels (CA-125 and HE-4) were extracted.

### 2.2 Data processing

**Figure 1:**
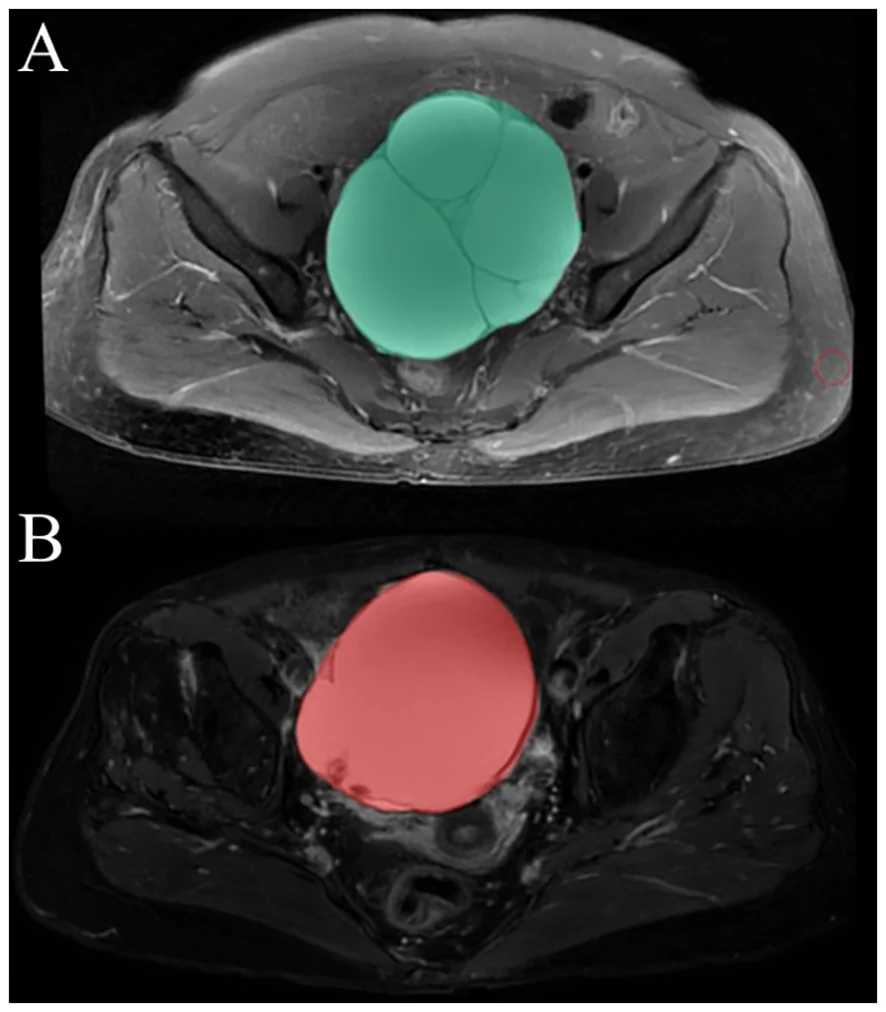
Segmentation examples. **(A)** Mucinous cystadenoma **(B)** Serous cystadenocarcinoma.

MRI scans were performed using 3.0T systems (GE HealthCare Signa). All the T2 MRI DICOM images and cancer biomarker levels (CA-125 and HE-4) were analyzed using uAI (United Imaging Intelligence, v20250130). Only the axial T2WI were retained, whereas the other sequences were discarded to avoid over-fitting. The axial T2WI images were individually examined, and manual segmentation of the ovarian lesions (region of interest (ROI)) were performed (**Figure 1**). The first 50 cases (randomly selected from the included patients) were segmented by two authors (a radiology resident and a radiologist with over 5 years of experience) independently and blinded to each other’s segmentations. We then performed a voxel-wise comparison of the segmentation masks and calculated the Dice similarity coefficient to assess interobserver agreement $\left(Dice=\frac{2\left|\text{A}\cap \text{B}\right|}{\left|A\right|+\left|B\right|}\right)$ using Python (NumPy library). The Dice similarity coefficient was very high (mean: 0.92, 95% confidence interval: 0.89-0.94). Radiomic features demonstrated excellent reproducibility (ICC >0.75 for the vast majority of retained features). Thus, because of high interobserver agreement and the nature of the study, we decided that the segmentations can be done by one observer and reviewed by the second observer with any differences eliminated after discussion. ROIs were selected according to the O-RADS MRI classification (20). All MRI slices underwent standardized preprocessing. Images were loaded as grayscale, resized to 256*256 pixels using bilinear interpolation, and intensity values were clipped to the 1st-99th percentile range to remove outliers. Min-max normalization was applied to rescale intensities to [0, 1].

### 2.3 Model development and evaluation

Model development and evaluation were performed in uAI. Only slices containing segmented tumor regions without cancer biomarker data were included in the initial model development. First, quantitative radiomics features were extracted. A total of 2286 features were initially generated for each case, including (1) first-order statistics describing voxel intensity distributions (e.g. mean, energy, entropy, kurtosis); (2) shape features capturing the three-dimensional morphology of the lesion (e.g. volume, surface area, compactness); (3) texture features characterizing spatial patterns and heterogeneity (e.g. Gray-Level Co-occurrence Matrix (GLCM), Gray-Level Run Length Matrix (GLRLM), Gray-Level Size Zone Matrix (GLSZM), and Neighboring Gray Tone Difference Matrix (NGTDM)); (4) wavelet-based features capturing both fine and coarse texture data. For descriptions of radiomics features, we recommend Maleki et al. (appendices) (27) for a detailed review and Zhang et al (28) for a brief overview.

The dataset was randomly divided into a training and testing set with a ratio of 4:1 (20% testing). To reduce dimensionality and prevent overfitting, a multi-step selection process was applied to the training set. First, variance thresholding was used to remove near-constant features leaving 2270 features. Next, features were screened using a univariate statistical ranking approach, retaining 100 top-ranking features. The intermediate selection of the top 100 variables provides sufficient candidate features while maintaining a favorable feature-to-sample ratio for the subsequent dimensionality reduction. The Least Absolute Shrinkage and Selection Operator (LASSO) regression was applied to further reduce the number of features, resulting in 37 final features with non-zero coefficients. The selected features were used to train machine learning models with different combinations of data scaling (min-max, Z-score, and quantile transformations) and classifiers (Logistic Regression (LR) and Partial Least Squares Discriminant Analysis (PLS-DA)). Thus, the following pairs were created: quantile-LR, quantile-PLSDA, Z score-LR, Z score-PLSDA, minmax-LR and minmax-PLSDA. A 5-fold cross-validation was performed for each model.

For the sub-study, only patients with available CA-125 and HE-4 were included. A model with the highest performance from the main study was used for training. The same imaging features identified in the main study were retained, and serum biomarkers were added to develop separate models. Thus, the following models were constructed: imaging + CA-125, imaging + HE-4, imaging + CA-125 + HE-4, and imaging alone (to allow direct comparison).

The performance of models was evaluated using sensitivity, specificity, accuracy, F1 score, and area under the receiver operating curve (AUC). For each model, the threshold corresponding to the maximum Youden Index was used. Performance differences between models were compared using DeLong’s method. At the maximum Youden threshold, differences in performance metrics were also assessed with McNemar’s test. P< 0.05 was considered statistically significant.

### 2.4 Statistical analysis

Statistical analysis was performed in R using the rstatix package. Normality was assessed using the Shapiro-Wilk test, and differences between groups were compared using an appropriate test (t test or Mann-Whitney U test). All values were recorded as mean ± SD (for normal distributions) or as median [Q1 – Q3] (for not normal distributions). Raw unprocessed radiomic features were recorded as mean ± SD. P< 0.05 was considered statistically significant.

## 3 Results

### 3.1 Radiomics features

**Figure 2:**
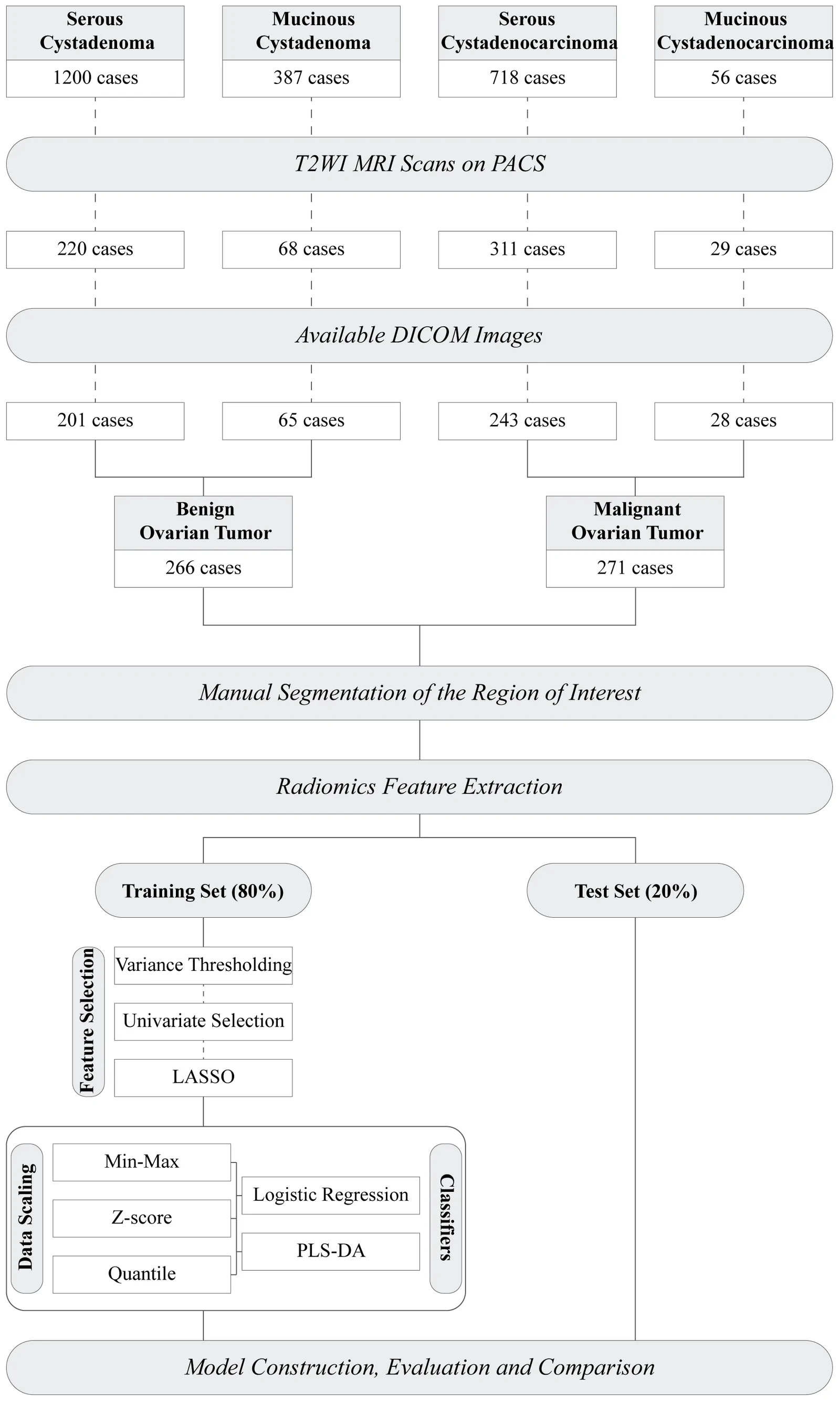
Flowchart of the study.

A total of 266 cases with cystadenoma and 271 cases with malignant cystadenocarcinoma were included in the study (**Figure 2**). The median age was significantly higher in the malignant group compared with the benign group (55 years vs 50 years, P< 0.001) (**Table 1**). The median volume of the ROI (tumor) was 228.51 [53.85–595.33] cm^3^ in the malignant group and 241.37 [77.48–645.66] cm^3^ in the benign group, with no statistically significant difference (P = 0.176). Finally, the median signal intensity of the ROI was significantly lower in the malignant group compared with the benign group (933.70 [643.46–1224.93] vs 1082.10 [668.78–1523.53], P< 0.001).

**Table 1:** Baseline characteristics.

Item	Malignant (n = 271)	Benign (n = 266)	P value
Age, years (n = 537)	55.0 [49.0 – 63.0]	50.0 [40.0 – 59.0]	<0.001
CA-125, U/mL (n = 197)	391.0 [59.0 - 1524.0]	23.0 [12.50 - 32.0]	<0.001
HE-4, pmol/L (n = 197)	289.0 [103.50 - 570.50]	39.0 [31.0 - 51.50]	<0.001
Volume of Region of Interest, mm^3^ (n = 537)	228512.58 [53853.22 – 595329.19]	241365.27 [77484.71 – 645662.29]	0.176
Intensity of Region of Interest (n = 537)	933.70 [643.46 – 1224.93]	1082.10 [668.78 – 1523.53]	<0.001

The majority of extracted radiomic features were significantly different between the two groups. Shapes of ovarian lesions differed: benign lesions were significantly “closer” to a spherical shape and had significantly greater elongation than malignant masses (0.71 ± 0.06 vs 0.66 ± 0.08, P< 0.001, and 0.75 ± 0.14 vs 0.72 ± 0.15, P = 0.010). Regarding texture metrics, malignancy was associated with significantly higher GLCM contrast (95.95 ± 116.92 vs 83.69 ± 134.41, P< 0.001), NGTDM contrast (0.21 ± 0.22 vs 0.18 ± 0.26, P< 0.001), GLSZM zone entropy (7.52 ± 0.51 vs 7.39 ± 0.79, P = 0.007) but significantly lower GLRM run entropy (5.89 ± 0.57 vs 6.03 ± 0.64, P = 0.015), GLDM dependence entropy (8.30 ± 0.63 vs 8.42 ± 0.82, P = 0.033). Detailed results for the raw unprocessed radiomics features are provided in **Supplementary Table 1**.

### 3.2 Model development using radiomics features

**Figure 3:**
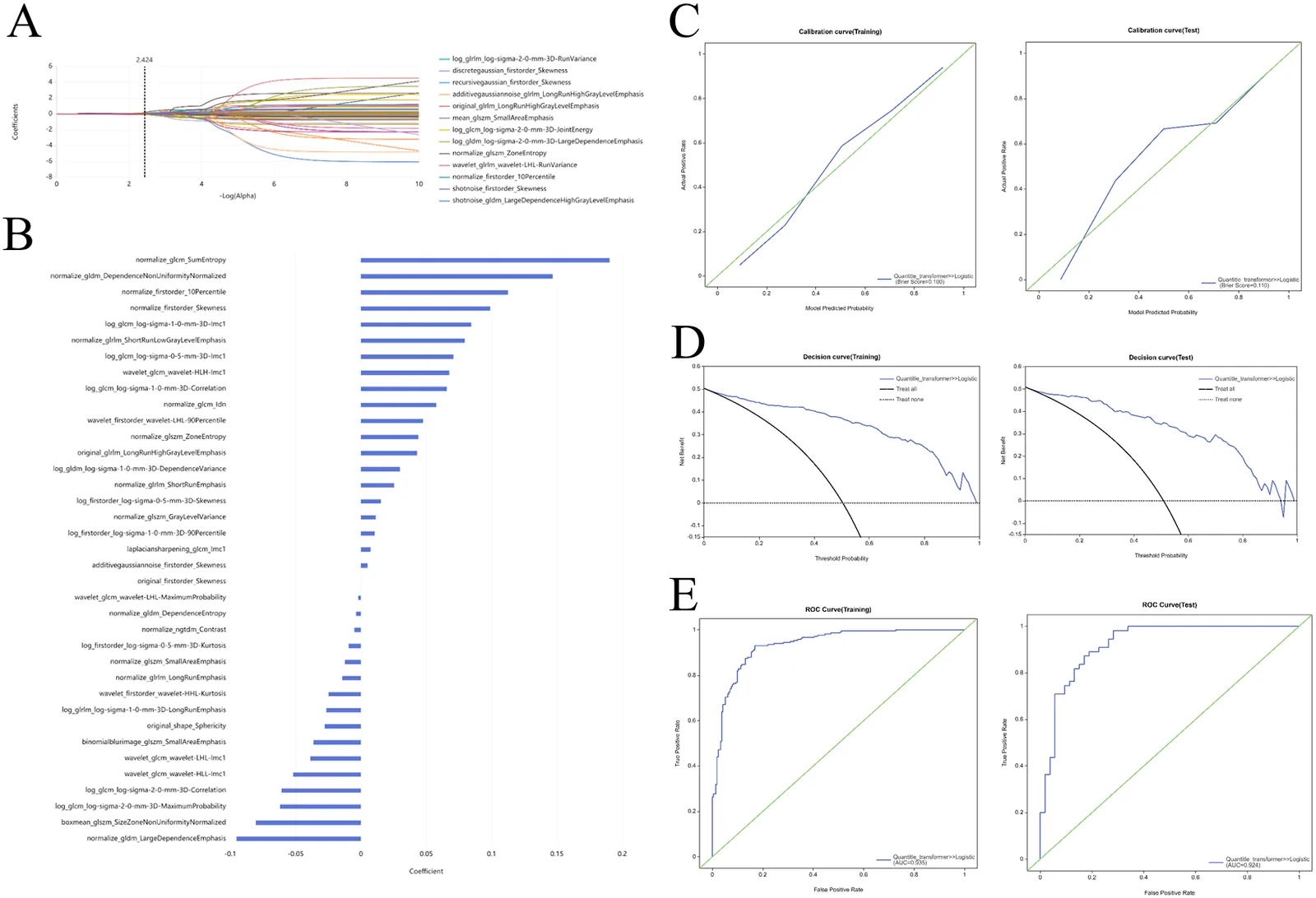
Model development **(A)** LASSO coefficient paths **(B)** Selected features by LASSO **(C)** Calibration curves (quantile logistic regression model) **(D)** Decision curve analysis curves (quantile logistic regression model) **(E)** Receiver operating characteristic curves (quantile logistic regression model.).

Following dimensionality reduction (variance thresholding, univariate statistical ranking, and LASSO), 37 features were identified and included for model development (**Figures 3A, B**). Among the evaluated machine learning models, the quantile-LR model exhibited the strongest overall performance. This model achieved an AUC of 0.92, sensitivity of 0.87, specificity of 0.83, accuracy of 0.85, precision of 0.84, and an F1 score of 0.86 in the test set (**Table 2**). Differences in models’ performance were assessed using DeLong’s test and McNemar’s test (**Table 3**). DeLong’s test revealed that the AUC of the quantile-LR model was significantly higher than that of quantile-PLSDA (p< 0.001), min-max PLSDA (p = 0.006), Z-score PLSDA (p = 0.003), and min-max LR (p = 0.011), whereas the difference in AUC compared to the Z-score LR model was not statistically significant (p = 0.247). Moreover, McNemar’s test showed that the quantile-LR model had significantly higher sensitivity, specificity and accuracy compared to all PLSDA models (all p< 0.01) but not compared to min-max LR and Z-score LR (both p > 0.05). The quantile-LR model demonstrated great probabilistic calibration and clinical utility, with a Brier score of 0.10 in the training set and 0.11 in the test set (**Figure 3C**). Decision curve analysis showed consistently positive net benefit, reaching a net benefit close to 0.3 at a 0.7 threshold (**Figure 3D**). Finally, as can be seen from the ROC curve, the model exhibited high predictive performance without falling below the random curve (**Figure 3E**).

**Table 2:** Performance of different radiomics-based models at maximum Youden Index threshold.

Performance metric	Min-max LR (0.6)		Mix-max PLSDA (0.62)		Z-score LR (0.59)		Z-score PLSDA (0.62)		Quantile LR (0.46)		Quantile PLSDA (0.61)	
Set	Train	Test	Train	Test	Train	Test	Train	Test	Train	Test	Train	Test
AUC	0.92	0.90	0.90	0.88	0.95	0.90	0.90	0.88	0.93	0.92	0.91	0.89
Sensitivity	0.80	0.85	0.86	0.87	0.84	0.82	0.86	0.87	0.88	0.87	0.86	0.84
Specificity	0.88	0.81	0.84	0.79	0.92	0.89	0.84	0.81	0.85	0.83	0.83	0.79
Accuracy	0.84	0.83	0.85	0.83	0.88	0.85	0.85	0.84	0.86	0.85	0.84	0.81
Precision	0.87	0.82	0.84	0.81	0.91	0.88	0.84	0.83	0.86	0.84	0.83	0.81
F1	0.83	0.84	0.85	0.84	0.87	0.85	0.85	0.85	0.87	0.86	0.87	0.82

LR, Logistic Regression; PLSDA, Partial Least Squares Discriminant Analysis; threshold is shown in brackets.

**Table 3:** P-values obtained from Delong’s and McNemar’s tests for compared radiomics-based models.

Model comparison	AUC	Sensitivity	Specificity	Accuracy
Min-max LR vs quantile LR	0.011	0.125	0.219	0.754
Min-max LR vs Z-score PLSDA	0.055	0.031	<0.001	<0.001
Min-max LR vs Z-score LR	0.763	0.625	0.125	1.0
Min-max LR vs quantile PLSDA	0.345	0.031	<0.001	<0.001
Min-max LR vs mix-max PLSDA	0.168	0.031	<0.001	<0.001
Z-score LR vs quantile PLSDA	0.485	0.008	<0.001	<0.001
Z-score LR vs mix-max PLSDA	0.371	0.008	<0.001	<0.001
Z-score LR vs Z-score PLSDA	0.237	0.008	<0.001	<0.001
Z-score PLSDA vs quantile PLSDA	0.360	1.0	0.688	0.031
Z-score PLSDA vs mix-max PLSDA	0.029	1.0	1.0	1.0
Quantile LR vs Z-score PLSDA	0.003	0.002	<0.001	<0.001
Quantile LR vs Z-score LR	0.247	0.625	1.0	0.688
Quantile LR vs quantile PLSDA	<0.001	0.002	<0.001	<0.001
Quantile LR vs mix-max PLSDA	0.006	0.002	<0.001	<0.001
Quantile PLSDA vs mix-max PLSDA	0.722	1.0	0.688	0.031

LR, Logistic Regression; PLSDA, Partial Least Squares Discriminant Analysis.

### 3.3 Model development using radiomics features and cancer biomarkers

A total of 197 patients (102 malignant tumors and 95 benign tumors) had lab results of CA-125 and HE-4 (**Table 1**). CA-125 and HE-4 were included in the model to test whether adding cancer biomarkers can improve the quantile-LR model’s performance. Inclusion of HE-4 or both CA-125 and HE-4 resulted in a significantly higher performance compared to the “original model” (P = 0.023 and P = 0.005, respectively), whereas no significant differences were observed between the performances of imaging + CA-125 and imaging alone models (P = 0.813) (**Table 4**).

**Table 4:** Performance of different radiomics + biomarker models (test set).

Model	AUC [95%CI]	Sensitivity	Specificity	Accuracy	Precision	F1 score
Imaging alone (A)	0.897 [0.790 - 1.0]^a^	0.857	0.842	0.850	0.857	0.857
Imaging + CA-125 (B)	0.982 [0.953 - 1.0]^a^	0.857	0.947	0.90	0.947	0.90
Imaging + HE-4 (C)	0.990 [0.968 - 1.0]^a^	0.857	0.947	0.90	0.947	0.90
Imaging + CA-125 + HE-4 (D)	0.980 [0.946 - 1.0]^a^	0.905	0.947	0.925	0.950	0.927

^a^ Pairwise AUC DeLong p-values.

A vs B: p = 0.813; A vs C: p = 0.023; A vs D: p = 0.005.

B vs A: p = 0.813; B vs C: p = 0.046; B vs D: p = 0.008.

C vs A: p = 0.023; C vs B: p = 0.046; C vs D: p = 0.044.

D vs A: p = 0.005; D vs B: p = 0.008; D vs C: p = 0.044.

## 4 Discussion

The objective of this study was to develop and evaluate radiomics models to distinguish benign from malignant cystic epithelial ovarian lesions on T2WI scans. We extracted quantitative imaging signatures from 537 patients and compared various machine learning models to identify the most reliable diagnostic workflow. Also, we tested whether the inclusion of cancer serum biomarkers (CA-125 and HE-4) could provide a statistically significant boost to the model’s predictive power.

We found that multiple extracted radiomics features were significantly different between benign and malignant ovarian lesions. Firstly, benign lesions showed significantly higher mean signal intensity and tended to be more spherical, which is consistent with observations made decades ago. Malignant ovarian lesions are associated with irregular shape and a lower average signal intensity due to necrosis, hemorrhage and other processes (29, 30). Notably, malignant and benign lesions were comparable in terms of entropy (shows how mixed the signal intensities are), which could be caused by an overlap, for example, between complex benign and early malignant lesions (31–33). Perhaps most importantly, the high-order textural features (e.g., GLCM and NGTDM contrasts, GLSZM zone entropy) provided a more detailed look into the structure of ovarian lesions than first-order statistics alone. Even though malignancies were associated with significantly lower signal intensity on average, they displayed significantly higher contrast and zone entropy (size-based heterogeneity). On the other hand, some entropy-based metrics (e.g., GLDM dependence entropy) were significantly higher in the benign group. Different radiomic features quantify different aspects of heterogeneity (e.g., GLDM dependence entropy measures the relationship between a voxel and its neighbors), so single metrics can be misleading when interpreted alone. Ultimately, such differences demonstrate the necessity of using multivariable radiomic models that take multiple features into account.

Radiomics models exhibited good performance for differentiating benign from malignant tumors across various cancer types, including breast tumors (34) and endometrial cancer (35, 36). Similar studies were conducted in differentiating ovarian tumors. A recent traditional descriptive study that relied on defined variables rather than modeling (37) reported very high diagnostic performance (AUC = 0.944). Other studies reported that CT- or MRI-based machine learning models are also associated with good performance, with reported AUCs typically between 0.82 and 0.90 for distinguishing benign, borderline, or early malignant ovarian tumors (38–40). However, most of the studies included a relatively small number of patients, which could result in overfitting and inflated performance. In comparison, our quantile-LR model achieved an AUC of 0.92, a sensitivity of 0.87, and a specificity of 0.83 using axial T2WI sequences alone. This performance is superior to other reported models, such as the T2-based radiomics model (specificity of 0.76, sensitivity of 0.82) and the CT-based ensemble model (specificity of 0.89, sensitivity of 0.68) (38, 39). Nonetheless, it must be noted that the inclusion and exclusion criteria in studies were different, as we focused only on epithelial cystadenomas and cystadenocarcinoma.

In our study, the initial radiomics feature set was reduced from over 2200 features to 37 features by implementing various feature selection tools. Next, we systematically evaluated several data scaling methods and machine learning algorithms. Quantile-LR significantly outperformed most other combinations in pairwise comparisons. The model showed excellent calibration, as evidenced by low Brier scores. This indicates that the model had low overall prediction error and good agreement between predictions and observations with minimal evidence of overfitting during internal validation (41). In decision curve analysis, which plots net benefit across a range of probabilities and compares a model’s decisions to two strategies: “treat all” (all patients are positive and receive treatment) and “treat none” (none are positive and receive treatment) (42) the quantile-LR model provided a higher net benefit than either of these two strategies. Although the addition of CA-125 resulted in a higher AUC compared with the imaging-only model (0.982 vs. 0.897), the difference was not statistically significant according to the paired DeLong test (p = 0.813). This is likely attributed to the relatively small differences between AUC values compared with the variability of the measurements (specifically, overlap in 95% CI of the two models; imaging alone: 0.790-1.000 and imaging + CA-125: 0.953-1.000). In addition, the compared models were correlated due to the use of the same imaging features. Finally, our results showed that combining imaging features with CA-125 and HE-4 biomarkers resulted in a significantly higher performance than using imaging data alone. This is consistent with prior studies, which showed that including clinical or laboratory data results in improved performance (34, 38, 40). Thus, the development and validation of multimodal models incorporating both imaging and tabular data is a promising future direction.

The present study has several limitations. First, this is a single-center study with a retrospective design. Although our model was trained on a balanced dataset with a substantial number of cases, achieved good results without any obvious evidence of substantial overfitting during internal validation, it was not externally validated. The lack of external validation may limit the generalizability of the constructed models to data acquired at other institutions using different MRI scanners and protocols. Radiomics features may be sensitive to variations in acquisition parameters, which may affect model performance. Second, acquisition parameters, such as voxel spacing, TR/TE (repetition time/echo time), and field of view, were not uniform among all patients, as this is a retrospective study that covered an eight-year period. Third, to keep the model “direct” and simple, only T2WI axial images were used. This excluded potentially valuable data from other sequences, such as T1-weighted scans, contrast-enhanced scans, etc. Fourth, the study was limited to the differentiation of epithelial cystadenomas and cystadenocarcinomas, and common non-epithelial tumors such as endometriomas and dermoid cysts were not included. Moreover, tumor staging was also not included in the analysis, which is extremely important in the clinical work, as the major diagnostic challenge usually lies in differentiating complex benign masses from early-stage malignant tumors. Finally, the model did not include major covariates, such as clinical presentation, menopausal status, hereditary history, etc.

## 5 Conclusion

The present study described the development of a “direct” model capable of differentiating between benign and malignant cystic epithelial ovarian lesions. The quantile-LR model exhibited the highest performance, achieving an AUC of 0.92, a sensitivity of 0.87, and a specificity of 0.83 in the test set. Finally, the inclusion of serum biomarkers (CA-125 and HE-4) significantly improved the performance of the model. However, as this is a single-center retrospective study, the generalizability of the constructed model is limited. The lack of external validation may have resulted in the overestimated reported performance. Thus, the findings should be interpreted as preliminary and hypothesis-generating rather than immediately clinically applicable.

## Data Availability

The raw data supporting the conclusions of this article will be made available by the authors, without undue reservation.

## Supplementary material

Supplementary Table 1 Raw unprocessed radiomics features.
