# Supplementary material for: Radiomics-based MRI model for differentiating ovarian cystadenoma and cystadenocarcinoma
Source: Front Oncol. 2026 Aug 27;16:1839964. doi: 10.3389/fonc.2026.1839964 (PMC13558202; doi:10.3389/fonc.2026.1839964)
Supplement: Supplementary file 1 [file Table1.docx]

# Supplementary Table 1 Original radiomics features

| Item | Malignant (n = 271) | Benign (n = 269) | P value |
| --- | --- | --- | --- |
| **First order** | | | |
| 10Percentile | 630.03 ± 295.96 | 841.45 ± 525.76 | <0.001 |
| 90Percentile | 1350.71 ± 621.33 | 1545.86 ± 809.23 | 0.019 |
| Energy | 127209492232.05 ± 224345569534.80 | 243928736452.41 ± 478457260992.08 | 0.004 |
| Entropy | 5.20 ± 0.73 | 5.06 ± 0.90 | 0.124 |
| InterquartileRange | 381.06 ± 241.40 | 360.46 ± 268.91 | 0.062 |
| Kurtosis | 3.82 ± 1.85 | 4.77 ± 2.72 | <0.001 |
| Maximum | 2079.17 ± 919.54 | 2137.41 ± 1055.07 | 0.731 |
| MeanAbsoluteDeviation | 228.25 ± 122.45 | 225.95 ± 143.07 | 0.334 |
| Mean | 973.30 ± 443.11 | 1200.18 ± 672.30 | 0.001 |
| Median | 954.33 ± 444.78 | 1210.55 ± 712.11 | <0.001 |
| Minimum | 107.79 ± 114.42 | 109.55 ± 158.03 | 0.182 |
| Range | 1971.38 ± 902.44 | 2027.86 ± 1045.47 | 0.803 |
| RobustMeanAbsoluteDeviation | 159.94 ± 96.81 | 153.73 ± 110.74 | 0.107 |
| RootMeanSquared | 1016.77 ± 461.49 | 1240.09 ± 683.80 | 0.001 |
| Skewness | 0.40 ± 0.71 | -0.34 ± 1.09 | <0.001 |
| TotalEnergy | 547442198646.91 ± 963794183065.54 | 1007397925551.60 ± 1979084679207.55 | 0.005 |
| Uniformity | 0.04 ± 0.02 | 0.05 ± 0.04 | 0.035 |
| Variance | 103072.98 ± 110115.34 | 112922.40 ± 138654.27 | 0.633 |
| **Shape** | | | |
| Elongation | 0.72 ± 0.15 | 0.75 ± 0.14 | 0.010 |
| Flatness | 0.54 ± 0.15 | 0.58 ± 0.15 | 0.002 |
| LeastAxisLength | 55.38 ± 26.99 | 59.41 ± 28.82 | 0.115 |
| MajorAxisLength | 105.43 ± 49.10 | 107.10 ± 56.29 | 0.753 |
| Maximum2DDiameterColumn | 108.27 ± 53.01 | 110.63 ± 60.39 | 0.876 |
| Maximum2DDiameterRow | 102.64 ± 51.64 | 107.94 ± 58.34 | 0.530 |
| Maximum2DDiameterSlice | 106.25 ± 49.25 | 107.89 ± 55.12 | 0.778 |
| Maximum3DDiameter | 120.48 ± 55.22 | 122.95 ± 62.69 | 0.962 |
| MeshVolume | 448481.33 ± 656129.71 | 589374.55 ± 894980.88 | 0.175 |
| MinorAxisLength | 74.82 ± 37.80 | 78.70 ± 43.20 | 0.508 |
| Sphericity | 0.66 ± 0.08 | 0.71 ± 0.06 | <0.001 |
| SurfaceArea | 37086.07 ± 33723.86 | 40429.52 ± 40405.37 | 0.729 |
| SurfaceVolumeRatio | 0.15 ± 0.09 | 0.14 ± 0.09 | 0.001 |
| VoxelVolume | 448960.82 ± 656347.81 | 589806.27 ± 895211.42 | 0.176 |
| **Gray-Level Co-occurrence Matrix (GLCM)** | | | |
| Autocorrelation | 1684.23 ± 1672.31 | 2821.69 ± 3541.84 | <0.001 |
| JointAverage | 36.04 ± 16.63 | 45.09 ± 25.88 | <0.001 |
| ClusterProminence | 1842915.11 ± 4484062.24 | 2826708.60 ± 7530504.73 | 0.771 |
| ClusterShade | 6594.23 ± 20036.71 | 864.34 ± 29813.97 | <0.001 |
| ClusterTendency | 534.35 ± 599.16 | 569.60 ± 793.69 | 0.267 |
| Contrast | 95.95 ± 116.92 | 83.69 ± 134.41 | <0.001 |
| Correlation | 0.67 ± 0.17 | 0.69 ± 0.22 | 0.028 |
| DifferenceAverage | 5.48 ± 3.28 | 4.38 ± 3.46 | <0.001 |
| DifferenceEntropy | 3.58 ± 0.73 | 3.15 ± 0.82 | <0.001 |
| DifferenceVariance | 47.71 ± 54.36 | 46.70 ± 69.69 | 0.033 |
| JointEnergy | 0.01 ± 0.01 | 0.01 ± 0.02 | <0.001 |
| JointEntropy | 9.13 ± 1.33 | 8.40 ± 1.57 | <0.001 |
| InverseVariance | 0.26 ± 0.07 | 0.30 ± 0.07 | <0.001 |
| MaximumProbability | 0.02 ± 0.02 | 0.03 ± 0.05 | <0.001 |
| SumEntropy | 6.01 ± 0.76 | 5.83 ± 0.96 | 0.048 |
| Idn | 0.94 ± 0.03 | 0.95 ± 0.03 | <0.001 |
| Id | 0.38 ± 0.11 | 0.46 ± 0.13 | <0.001 |
| Idmn | 0.99 ± 0.01 | 0.99 ± 0.01 | <0.001 |
| Idm | 0.31 ± 0.11 | 0.40 ± 0.15 | <0.001 |
| Imc2 | 0.87 ± 0.09 | 0.92 ± 0.08 | <0.001 |
| Imc1 | -0.22 ± 0.07 | -0.30 ± 0.09 | <0.001 |
| **Gray-Level Run Length Matrix (GLRLM)** | | | |
| GrayLevelNonUniformity | 2675.88 ± 3534.45 | 3256.43 ± 3887.26 | 0.122 |
| GrayLevelNonUniformityNormalized | 0.04 ± 0.02 | 0.04 ± 0.03 | 0.132 |
| GrayLevelVariance | 168.35 ± 175.25 | 190.21 ± 224.18 | 0.967 |
| HighGrayLevelRunEmphasis | 1691.48 ± 1647.78 | 2799.42 ± 3404.51 | <0.001 |
| LongRunEmphasis | 2.28 ± 1.48 | 4.43 ± 6.57 | <0.001 |
| LongRunHighGrayLevelEmphasis | 3146.32 ± 3200.78 | 6701.76 ± 6786.60 | <0.001 |
| LongRunLowGrayLevelEmphasis | 0.01 ± 0.04 | 0.02 ± 0.06 | 0.704 |
| LowGrayLevelRunEmphasis | 0.00 ± 0.00 | 0.00 ± 0.00 | 0.012 |
| RunEntropy | 5.89 ± 0.57 | 6.03 ± 0.64 | 0.015 |
| RunLengthNonUniformity | 62588.18 ± 73225.47 | 73476.59 ± 107794.46 | 0.998 |
| RunLengthNonUniformityNormalized | 0.78 ± 0.09 | 0.71 ± 0.13 | <0.001 |
| RunPercentage | 0.85 ± 0.07 | 0.78 ± 0.11 | <0.001 |
| RunVariance | 0.66 ± 0.89 | 2.01 ± 4.29 | <0.001 |
| ShortRunEmphasis | 0.90 ± 0.05 | 0.85 ± 0.08 | <0.001 |
| ShortRunHighGrayLevelEmphasis | 1550.84 ± 1550.21 | 2476.34 ± 3040.56 | 0.002 |
| ShortRunLowGrayLevelEmphasis | 0.00 ± 0.00 | 0.00 ± 0.00 | 0.001 |
| **Gray-Level Size Zone Matrix (GLSZM)** | | | |
| GrayLevelNonUniformity | 396.19 ± 370.44 | 288.99 ± 284.39 | 0.002 |
| GrayLevelNonUniformityNormalized | 0.03 ± 0.01 | 0.03 ± 0.02 | 0.020 |
| GrayLevelVariance | 190.92 ± 175.26 | 252.53 ± 264.47 | 0.030 |
| HighGrayLevelZoneEmphasis | 1663.21 ± 1518.87 | 2531.20 ± 2818.20 | 0.003 |
| LargeAreaEmphasis | 20584.42 ± 85293.53 | 80444.24 ± 306104.85 | <0.001 |
| LargeAreaHighGrayLevelEmphasis | 11909630.71 ± 53622445.69 | 41515690.97 ± 85644397.63 | <0.001 |
| LargeAreaLowGrayLevelEmphasis | 139.47 ± 815.35 | 460.98 ± 2440.22 | <0.001 |
| LowGrayLevelZoneEmphasis | 0.00 ± 0.01 | 0.00 ± 0.00 | 0.692 |
| SizeZoneNonUniformity | 7254.24 ± 8491.42 | 6519.57 ± 8204.07 | 0.343 |
| SizeZoneNonUniformityNormalized | 0.40 ± 0.08 | 0.46 ± 0.09 | <0.001 |
| SmallAreaEmphasis | 0.66 ± 0.07 | 0.70 ± 0.07 | <0.001 |
| SmallAreaHighGrayLevelEmphasis | 1141.65 ± 1119.25 | 1735.22 ± 2032.30 | 0.002 |
| SmallAreaLowGrayLevelEmphasis | 0.00 ± 0.00 | 0.00 ± 0.00 | 0.733 |
| ZoneEntropy | 7.52 ± 0.51 | 7.39 ± 0.79 | 0.007 |
| ZonePercentage | 0.26 ± 0.15 | 0.19 ± 0.14 | <0.001 |
| ZoneVariance | 20502.47 ± 85044.24 | 80244.20 ± 305406.62 | <0.001 |
| **Gray-Level Size Zone Matrix (GLDM)** | | | |
| DependenceEntropy | 8.30 ± 0.63 | 8.42 ± 0.82 | 0.033 |
| DependenceNonUniformity | 10224.75 ± 11617.37 | 11159.77 ± 16093.19 | 0.439 |
| DependenceNonUniformityNormalized | 0.14 ± 0.07 | 0.10 ± 0.06 | <0.001 |
| DependenceVariance | 11.22 ± 7.88 | 18.25 ± 12.50 | <0.001 |
| GrayLevelNonUniformity | 3576.70 ± 5303.30 | 5185.47 ± 7482.00 | 0.015 |
| GrayLevelVariance | 165.00 ± 176.18 | 180.76 ± 221.85 | 0.632 |
| HighGrayLevelEmphasis | 1694.10 ± 1664.16 | 2804.74 ± 3468.46 | <0.001 |
| LargeDependenceEmphasis | 38.36 ± 29.08 | 68.71 ± 56.18 | <0.001 |
| LargeDependenceHighGrayLevelEmphasis | 48218.56 ± 58802.56 | 117736.56 ± 144679.01 | <0.001 |
| LargeDependenceLowGrayLevelEmphasis | 0.15 ± 0.41 | 0.24 ± 0.56 | 0.370 |
| LowGrayLevelEmphasis | 0.00 ± 0.00 | 0.00 ± 0.00 | 0.005 |
| SmallDependenceEmphasis | 0.24 ± 0.13 | 0.18 ± 0.12 | <0.001 |
| SmallDependenceHighGrayLevelEmphasis | 493.73 ± 689.61 | 557.09 ± 790.43 | 0.910 |
| SmallDependenceLowGrayLevelEmphasis | 0.00 ± 0.00 | 0.00 ± 0.00 | 0.006 |
| **Neighboring Gray Tone Difference Matrix (NGTDM)** | | | |
| Busyness | 3.47 ± 7.20 | 3.30 ± 6.61 | 0.021 |
| Coarseness | 0.00 ± 0.01 | 0.00 ± 0.00 | 0.368 |
| Complexity | 15853.39 ± 24689.06 | 15080.70 ± 24775.53 | 0.138 |
| Contrast | 0.21 ± 0.22 | 0.18 ± 0.26 | <0.001 |
| Strength | 2.37 ± 13.72 | 2.27 ± 5.92 | 0.244 |
